# The After-Care of Obstetrical Patients

**Published:** 1906-03

**Authors:** Stephen T. Barnett

**Affiliations:** Atlanta, Ga., Surgeon Presbyterian Hospital


### THE AFTER-CARE OF OBSTETRICAL PATIENTS.*

By STEPHEN T. BARNETT, M.D., ATLANTA, GA.,
Surgeon Presbyterian Hospital.

Under this heading the care of both mother and infant must be considered during the period from the expulsion of the placenta to the return of the woman to the normal condition. Many of the things that I will speak of properly belong to the conduct of labor itself though technically they may be considered as in the puerperium. This after-treatment is most important, though there is a feeling among many that it is more or less trivial and many practitioners are prone to let nature take care of herself. But a proper care at this time will often mean immunity from ill-health in after-years if not actually preserving life itself.

The first care after the birth of the placenta should always be towards seeing that the uterus is contracting properly, and thus avoiding possible post-partum hemorrhage. I always make it a point to personally examine the organ at once to see how hard it is, and then if possible to place some one there to watch it with instructions to either kneed it until it returns to normal or to notify me if the softening continues. Oftentimes I find it a most accept-

* Read before the Fulton County Medical Society.

able place for some well-meaning but bothersome member of the family who is constantly putting her hands where they ought not to be. It should be watched carefully for the first hour, longer if necessary. Post-partum hemorrhage usually manifests itself first by the organ softening before the actual flow of blood, and if attention is at once given, can very easily be controlled as a rule, by kneeding and pinching or massaging the uterus. Before giving anything by mouth I always satisfy myself as to whether the placenta is intact. For it is much better to clean the uterus out at once without the drug adding to the difficulty. If the cleaning out is done I use the greatest precautions possible, the placenta forceps and the dull curette with sterile water or normal salt solution as hot as possible. Sometimes it is best to use the fingers and when I do I prefer the use of gloves. Occasionally if there is reason to think that there has been an infection it is wiser to use a bichlor. sol. 1-10000. If there is no need of cleaning out the womb, and there is usually not, but the bleeding continues after kneeding I give from fifteen to thirty drops of ergotole, usually by mouth though it can be given by hypo.

The next step is usually the repair of lacerations if any. Some advocate making the repair during the third stage, or at least placing the sutures at that time. I think that this is often perfectly feasible, though I have not myself ever done so. The advantages are that you can continue this operation with the same anesthesia as the labor and the subsequent tying of the sutures after the labor does not give much pain. I believe that this repair, if not done in the third stage, should always be done as soon after labor as possible, and done under the most stringent asepsis or antisepsis that the circumstances of the case permit. The examination for these tears should be carried on carefully and with a good light as, unless one's experience is good, it is not hard to overlook even severe tears owing to the general contused conditions of the parts. Nothing is more ludicrous to the gynecologist than to hear some youthful obstetrician proclaim the fact that he has never had a tear, though he has had so and so many cases, or to hear another say with self-laudatory mien, that he used to have tears but he has learnt how to handle them now, and that in the last one hundred cases he has not had a single tear. Of course the gynecologist later has to do a little repair work, and has to almost swear his soul into purgatory, trying to protect his brother who had assured his patient that she had not the semblance of a tear. Unfortunately this same statement is made by older men who have either continued in the way of their youth, unable to recognize a laceration when they see it, or who are without the manhood to admit that they are on all occasions able to prevent them. They occur to all, and in from ten per cent. to fifty per cent. of all cases as they run. The repair, as I have already said, should always be done. This in those of the third class as well as the first or second. Failures will occasionally result especially in those of the third degree. I believe that failures are often attributable to the fact that the sutures have been tied too tight. I am sure that some of my failures have been due to that alone. The subsequent edema is great and throws a great tension on the stitches and they will frequently cut through. Sometimes, of course, it is due to the fact that the woman is infected or you yourself have been negligent in some detail of your asepsis. I prefer silkworm-gut, though I have used catgut when the tears are superficial.

Having completed the repair work, if needed, the toilet of the vulva should next be taken up. A bichlor. cloth is placed over the vulva while the other parts cleansed by the nurse and then the surgeon himself cleans the vulva and the site of the stitches, if any, with a solution bichloride 1 to 2000. If the sponges come in contact with the rectum they should be thrown away and fresh ones taken. It is well always to sponge from before back and down. The vaginal douche, I think, unwise and I do not use it. A sterile gauze pad is then placed over the vulva. This is renewed as occasion demands, but always with a due regard to asepsis. This having been completed the soiled gown and linen is changed and the mother made comfortable, the room darkened and she is allowed to sleep, if possible. The nurse should even then be cautioned to watch out for hemorrhage. After the first few days the discharge or lochia commences to change in character, becoming paler and getting less in quantity. I have had cases in which it stopped on the second day, but these are unusual. The character of the lochia is important, often being the first sign that we have that an infection has taken place. It becomes foul in odor and more like pus. As a rule the temperature also goes up. If the odor is very bad and the temperature gets above 100.5 I give a vaginal douche of bichlor. 1 to 5000 at about 105°F. and use a long curved perforated glass nozzle with the bag at moderate elevation and using from two to six quarts of the solution. This is repeated from one to two times a day until the temperature drops and the discharge is less. Occasionally owing to the high and persistent fever intra-urine douches are necessary. I never use a stronger solution here than 1 to 10000 bichloride. It is almost needless to add that both these douches should be given under the strictest antiseptic precautions.

The nipples should have treatment beforehand if retracted. If tender they can be bathed with alcohol, or a tannic acid solution. Occasionally they are so tender that nipple shields have to be used or the breast pump. Cracks should be carefully watched for. If they occur I use tn. benzoin comp. to paint them and have found this more satisfactory than the solutions of nitrate of silver. The nipples should be bathed both before and after nursing with a solution of boric acid.

These precautions are very important, as if negligence here is allowed an abscess of the breast may be the penalty. The breasts may become caked and if the proper care is not given will abscess. These cakes should be carefully massaged and hot applications made which will generally reduce it. Some have found that the use of pressure has been useful in these cases. If, despite treatment, the abscess occurs, open at once, that is, if it is determined that it is there, and on the lower side, washing out each day with dioxygen or normal salt solution until it heals. The child should be taken from the breast, or both of them if it shows signs of being affected by the infection.

Immediately after labor there is usually retention of urine for awhile. I believe that in the voiding of it and of the bowels as well, that it is best, and in fact, is good that they do so in the sitting posture. It is certainly far preferable to the passage of the catheter and not as liable to infection. After the voiding the parts are rinsed with 1 to 2000 bichlor. sol. and a fresh pad put on. It gives the vagina an opportunity to throw off some clots that otherwise may stay in it quite a long time. The bowels should be kept open, and I prefer castor oil. Of course if the tear is of the third degree the bowels are not opened for a day or so, preferably.

The temperature is generally an indication that some infection has taken place, or, that the bowels need opening. I usually disregard it if below 100.5 as there is undoubtedly an obstetrical fever that is perfectly normal.

In the matter of diet I am more liberal than I used to be. I give eggs and tea and toast the first day and then gradually increase it in four or five days to light diet that is easily digested. I think that too often in the past we starved these mothers. This is, or ought to be, a physiological, not a pathological condition.

When I began to practice I was taught that the binder was very essential. That it preserved the figure, that it kept down the after-pains, and that it hastened involution. With the exception of the after-pains which it will occasionally relieve, this is a fallacy. If the tone of the abdominal muscles is lost no amount of binding is going to restore the figure. This can best be attained by massage in some form. Using pressure as it does in front of the distended uterus it tends to force it back into the retroverted position, to cause an increase in the pelvic pressure, and hence a retarding of the return circulation, which adds to the weight of the uterus, and a consequent subinvolution. I have discontinued the use of the binder. For the after-pains I use gentle massage and position. The matter of posture I consider very important. The woman should not be allowed to remain continuously on her back; she should be directed to change frequently and also that she should for a certain time each day lie on her abdomen. In this way it is possible to take the weight off of the ligaments and promotes involution. I believe that a steady persistence in our former methods of keeping our cases on their backs has been responsible for many of the retrodisplacements that we see as gynecologists. I am perfectly persuaded that this method is conducive of a quicker and a more thorough involution. They are generally perfectly willing to do so unless they have tender breasts.

The getting up time has for a long time been considered to be the tenth day. I do not believe that any fixed time should be made, as individual cases sometimes require a longer period to be fit to be about continuously and I have seen those that got up on the fifth and sixth days and not have any ill effects apparently. Of course the answer to so soon getting up is always that it is impossible to tell what the after-effects may be, and that unless these cases can be followed for a long period it would hardly 'establish the fact that there was not permanent injury. Personally I follow the plan of letting them get up as soon as the fundus is not felt above the symphysis. This is rarely before the tenth and sometimes not before the fourteenth day. In getting up, I think that it is important that they are not up too long the first few times. I know of their having been allowed to get up the first time on the tenth day and stay up all afternoon and the next day to be up the whole time. This is a mistake. These cases should be allowed to get up slowly. The first time from ten to fifteen or twenty minutes. This I let them repeat again in an hour or so. The next day they stay up for a half an hour to a full hour, depending on their strength and the severity of the labor. I always insist that they lie down even at the end of the month for sometime each day and if they are exhausted that they lie down frequently. It is folly to try to treat all these cases alike, and have arbitrary times for doing this or that.

One of the greatest essentials of any person more or less confined is plenty of fresh air. The old way of keeping the room dark and shut up is absolutely wrong. Nobody can thrive or get strong breathing and rebreathing the same foul air. In addition the baby needs it. I believe in fresh air and a sunny room for these women, and am sure that they improve faster than being cooped up.

If stitches have been placed I generally remove them on the tenth to twelfth day, as by that time they have served their purpose and if there is to be union it will have taken place. It should be done under aseptic conditions. I have been using for some time for the purpose of cutting these sutures a long-shanked, short-bladed scissors with a hook on one blade which I place under the suture and being sure that I have what I want and no tissue, can readily cut it out. It has, of course, been used for some time by many others. It facilitates the removal and with so little pain, that I have been constrained to mention it.

Finally, a last examination should always be made to ascertain just the condition of affairs and not allow yourself to be mortified perhaps at a later time by some other examiner finding what you should have found and corrected, or if not allowed to do so to have told her what it was. Some time it is so strenuously objected to that it is impossible to do it.

In regard to the baby, first see to the eyes, cleaning them with boric acid solution, or if there is a suspicion of gonorrhea it is well to follow Crede's method of dropping a drop of nitrate of silver solution in each eye. The mouth should be cleansed out with boric solution. These spongings off should be done with great tenderness. I think that oftentimes we are too rough, forgetting the delicacy of the tissues.

If the child does not promptly begin to breathe some method as of slapping or sprinkling with cold water or blowing or placing in cold and then warm water should be used. If not, then artificial respiration.

We should always make a careful examination for any deformities or anything unusual and tell the parents. It is their right and will save you some embarrassment probably in the future. At this same time it is well to measure and weigh the infant and keep a record of it.

The cord I cut about an inch long and have found some advantage in milking out some of Wharton's jelly before tying. I use preferably sterile starch or talcum powder to dust around it and then put on the binder. I believe it is a mistake to use most of the prepared cord powders, as they have acetanilid or some other drug which seems to preserve the cord. I had one case that it stayed on for fifteen days, much to my annoyance and the worry of the mother. Another friend of mine had one to stay on twenty-one days. I think that his was due to too much cleaning and the cord was actually kept from becoming detached by cleanliness. So I often do not touch mine for two or three days and then only to look at it and add more powder. I have so far had no trouble with infection and believe that too much handling is wrong.

I grease the infant with lard or vaseline and do not let the bath be given for twelve to twenty hours, as the vernix caseosum comes off more easily by so waiting than if washed at once.

The infant should sleep from twelve to twenty hours a day at first, gradually decreasing. They should be warmly clad, and in winter time if not put in the bed with the mother should be carefully protected from getting chilled in its crib. I have frequently seen infants that cried all night simply because they were suffering with cold and as soon as put next to the mother or the bed warmed would drop off to sleep. Because they are taken into the bed with the mother and generally do nurse it is generally thought that it is because they were hungry when it might not be the case at all.

They should nurse every two hours during the day and at night should go for an interval of three or four hours as a rule. It is wonderful what good habits can be so quickly learned by these little ones and what great discomfort bad ones give. The beauty of such training is seen to best advantage in lying-in hospitals where the mothers and infants are controlled and the effects speak for themselves.

The bowels and kidneys are usually active enough. Two to four times at the beginning are not too many actions to have. Occasionally, owing to some fault with the milk, they are constipated. That really falls under the head of pediatrics and must be treated along those lines. In the use of a cathartic I prefer castor oil.

I can not close this without a protest to the common practice of shaking or jostling the infant about. I think that this practice very often results in the child becoming nervous, irritable and often to lose in strength and health.

### DISCUSSION OF DR. BARNETT'S PAPER.

*Dr. Crowe*: I wish to thank Dr. Barnett for his excellent and sensible paper. It is of interest to all practitioners except perhaps the eye, ear, nose and throat men, for I guess all of us attend some obstetrical cases at times. That which stands first in importance in treatment of these cases is to remember we have a physiological and not a pathological condition to deal with. In all other cases or conditions we are called on to care for there is more or less the element of pathology. There are three fixed principles we should always remember: (1) cleanliness; (2) healthy sanitary surroundings; (3) absence of meddlesome interference. Nine-tenths of the cases of puerperal fever are due to infection introduced from without; for that reason I am very careful of the nurse engaged for these cases. No matter how careful the doctor has been, an incompetent nurse can undo all. I think we should do away with the untrained negro nurse.

I give a dry diet for first three or four days. Very little fluid, and in that way I can control the breast and coming of milk. I used to give the liquid diet *only* for first few days and frequently had trouble with breast. I have had very little trouble since giving only dry diet. I am opposed to the douche for first ten days; after that I use it for cleanliness. I do not approve of carbolic acid used in douche. Others may get good results from its use, but there are so many susceptible to carbolic acid poison that I do not feel justified in using it. I never use ergot except in case of hemorrhage. I use it in post-partum hemorrhage.

I do not, as a rule, attempt to repair a lacerated cervix immediately after labor. I have never performed the operation at such a time except in one or two cases. The tissues are in such condition that we are only justified when the walls are split up to the circular artery. I always repair the perineum at once. I use a running suture No. 1 plain catgut for mucous membranes. For the outside I use No. 2 chromic catgut and it does just as well as silkworm-gut. Every man should do a thing because he has a reason for it. That is where we get our individuality.

Now, I have used an ointment with base of lanolin combined with some antiseptic on skin incision. Have got better results since using it—can not say why. It may be only my improvement in technique. I am sure there is no operation in which unsatisfactory results are more often obtained. I have seen them where the skin was only closed, but no perineal support. If I have a trained nurse I catheterize the patient for several days after the operation. If no trained nurse I make my patient get in the knee-chest position to void her urine. I have seen as profound shock after a case of labor as in abdominal section. In case of shock I use morphine and strychnine. If there is any temperature after shock I consider it due to infection, except possibly that temperature which is due to the breast. Now, as to the nipple, there is nothing, in my opinion, which can take the place of lanolin. The dress of the patient during pregnancy—that is a tight shirt or vest—causes the fissure. I generally instruct my patient as to the proper mode of dress. I put the child to breast just after labor. If you neglect to do this for three or four days you will find trouble in teaching the babe to nurse. As to tying the cord. If I have a tedious labor, I wait until the cord stops pulsating—sometimes have to wait ten minutes. I never curette unless I am certain there is something in the womb. I wash out with normal salt solution. I agree with Dr. Kime in his method of draining, which he advocated years ago.

*Dr. Kmie:* I agree with the essayist in most all points presented. I do not introduce perineal stitches before delivery of placenta, because not so aseptic. I use No. 2 plain catgut in sulci, taking up vaginal mucosa and lacerated tissues beneath in a continuous suture; use prepared silk; skin surface of perineum. I place sterile silk in a mixture of one part camphorated phenol to two parts juniper oil, or pure olive oil, which renders it non-absorbing and gives good results in primary perineal repair.

I do not use vaginal douches until infection occurs, except in instrumental deliveries, then an intra-uterine douche is given immediately after delivery. When infection occurs, vaginal douches are first given, then intra-uterine douches followed with gauze and tubal drainage. I have condemned the use of curette for years in septic cases following labor near full term. Its use is dangerous, opens up new avenues of infection, breaks down nature's barriers to the invasion of infection and kills more than it saves. I use a curved sponge holding forceps to remove foreign material in uterus. Mild antiseptic douches are best, least irritating and less dangerous. Boric acid, salt solution or plain sterile water are best.

I wish to emphasize the question of position of the puerperal woman. I advised the exaggerated Sims position twelve to eighteen hours out of the twenty-four in obstetric work for years. Such a position favors drainage of uterus, prevents accumulation and retention of clots in vagina that would occur when kept on back, hastens involution and helps to hold the womb forward until the natural supports regain their tone.

In cases of retroversion of uterus appropriate treatment and support following the puerperal period after labor and abortion will permanently relieve many cases without operative measures.

*Dr. Boynton:* The ground has been so well covered that I mention only a few points which have been of comfort to me. I was taught that if a temperature came on the third day or the lochia slowed up, woe be unto your patient. I have long since ceased to worry. If temperature comes on third day there is no necessity of alarm. It may be caused from the breast or the bowels. If there is a decrease of the tenderness and no odor to the lochia, the temperature is caused by some other cause than uterine infection.

*Dr. Duncan:* I am opposed to the use of medicated douches. I use simply sterilized hot water, not only in the vagina, but uterus. There is one point I would like to mention in regard to abscess of breast. I simply open them and allow the pus to drain. I have tried washing out the abscess cavity with peroxide hydrogen but found result unsatisfactory.

*Dr. Cartledge:* Without taking the time to say the value the paper and discussions have been to me, I'll just mention three points I've thought valuable or useful in obstetrical work. I have resuscitated the asphyxiated new born by freely divulsing the sphincter ano when all other efforts failed. (2) Sweet oil with ordinary gauze cloth most effectually removes the vernix caseosum. (3) Solution of potassium permanganate to child's eye I have thought much preferred to silver or boric solution.

*Dr. Andrews:* As regards the treatment of puerperal infections a procedure has been devised of recent years, from which I have seen very good results; and results which are superior to those obtained by the method of irrigation. A hole is cut opposite the eye of a small rubber catheter, and through this is passed a piece of gauze and wrapped around the end of the catheter. This is placed in the uterus, with opposite end protruding from the vagina, one drachm of fifty per cent. alcohol is injected through this catheter every hour, taking care to force, with air, all of the alcohol out of the catheter into the gauze. Fifty per cent. alcohol is an efficient antiseptic, being a much greater one than ninety-five per cent. Alcohol has a great affinity for water, and the water which is extracted from the uterine tissue, in this way does good. Further than that, free drainage is established around the catheter, and this certainly does good in keeping the uterus clean.

*Dr. Clarke:* The paper read by Dr. Barnett is an excellent one, and in the main I have always attempted to follow such plans in practice. However, I disagree in one or two matters—ergot in post-partum hemorrhage—I certainly would not care to depend upon doctor's idea of twenty to thirty drops of ergotole in impending post-partum hemorrhage. I have had some exciting experiences with such cases, and where I have any attempts at bleeding from a uterus I know to be empty, I do not count drops or use mouth but give a syringe filled with ergot hypodermically. I do not believe that ergotole possesses any value over Squibb's fluid extract, which I generally use.

Again, I fail to see the disadvantage existing in the umbilical cord remaining on for ten to fourteen days. The case of twenty-one days was rather long, but certainly unusual. On the other hand, I can see no advantage in having the cord drop off in four or five days. I use boric acid in dressing my cords, and do the work myself. True the dressing acts as a preservative in addition to an antiseptic, and the cords usually remain on ten to even fourteen days, average ten, but they come off as a shriveled string, leaving a clean healed stump, and never any raw or bleeding surface, or as I have seen, pus. An important consideration is tetanus, which usually begins at the time of separation of the cord, and the site of infection is the umbilical stump. Nine days being the average limit for possible infection, my plan of preserving the cord protects against the disease.

The cord without an antiseptic simply rots off, and I do not think this should occur.

Lastly, I agree with Dr. Kime, that bichloride of mercury has no place in the uterine cavity.

*Dr. Barnett* (in closing): I heartily agree with the gentlemen in regard to the negro nurse, and I hope the time will soon come when we can do away with the untrained negro. I am opposed to the use of chromic catgut. I find it irritates, and on several occasions, have had bloody lymph to form from its presence in the skin. In regard to the use of the catheter, will say I do not think it should be used, as it is impossible to thoroughly cleanse and render aseptic the area around the meatus.

Unless the temperature rises over 101 F. I do not consider it advisable to give douche. After ten or twelve days, when the parts have healed, I use douche of hot salt solution. I have seen cases in which salt or boracic intrauterine douches have failed to bring the temperature down and bichloride relieved. I never use bichloride douche stronger than 1 to 10000 in the uterus.

There is no need of putting babe to breast so far as food is concerned, but it does have a beneficial effect by causing the uterus to contract.

In regard to the method of Dr. Andrews, I believe the good resulted from the drainage effected. Dr. Clarke misunderstood my remarks in regard to ergotole. I did not mean to convey the idea that twenty to thirty drops of ergotole would control post-partum hemorrhage, but if there is a tendency to bleeding which is not easily controlled by Crede's method, I am confident by personal experience that a small dose of ergotole by mouth is often all that is required, though, of course, if the bleeding is more persistent or larger in quantity it will require a larger and more quickly acting dose.

